# The NOURISH randomised control trial: Positive feeding practices and food preferences in early childhood - a primary prevention program for childhood obesity

**DOI:** 10.1186/1471-2458-9-387

**Published:** 2009-10-14

**Authors:** Lynne A Daniels, Anthea Magarey, Diana Battistutta, Jan M Nicholson, Ann Farrell, Geoffrey Davidson, Geoffrey Cleghorn

**Affiliations:** 1 School of Public Health, Institute of Health and Biomedical Innovation, Queensland University of Technology, Brisbane, Australia; 2 Nutrition and Dietetics Department, School of Medicine, Flinders University, Adelaide, Australia; 3 Institute of Health and Biomedical Innovation, Queensland University of Technology, Brisbane, Australia; 4 Murdoch Children's Research Institute and Centre for Learning Innovation, Melbourne, Australia; 5 Queensland University of Technology, Brisbane, Australia; 6 School of Early Childhood, Queensland University of Technology, Brisbane, Australia; 7 Centre for Paediatric Gastroenterology, The University of Adelaide, Adelaide, Australia; 8 School of Medicine, The University of Queensland, Brisbane, Australia

## Abstract

**Background:**

Primary prevention of childhood overweight is an international priority. In Australia 20-25% of 2-8 year olds are already overweight. These children are at substantially increased the risk of becoming overweight adults, with attendant increased risk of morbidity and mortality. Early feeding practices determine infant exposure to food (type, amount, frequency) and include responses (eg coercion) to infant feeding behaviour (eg. food refusal). There is correlational evidence linking parenting style and early feeding practices to child eating behaviour and weight status. A focus on early feeding is consistent with the national focus on early childhood as the foundation for life-long health and well being. The NOURISH trial aims to implement and evaluate a community-based intervention to promote early feeding practices that will foster healthy food preferences and intake and preserve the innate capacity to self-regulate food intake in young children.

**Methods/Design:**

This randomised controlled trial (RCT) aims to recruit 820 first-time mothers and their healthy term infants. A consecutive sample of eligible mothers will be approached postnatally at major maternity hospitals in Brisbane and Adelaide. Initial consent will be for re-contact for full enrolment when the infants are 4-7 months old. Individual mother- infant dyads will be randomised to usual care or the intervention. The intervention will provide anticipatory guidance via two modules of six fortnightly parent education and peer support group sessions, each followed by six months of regular maintenance contact. The modules will commence when the infants are aged 4-7 and 13-16 months to coincide with establishment of solid feeding, and autonomy and independence, respectively. Outcome measures will be assessed at baseline, with follow up at nine and 18 months. These will include infant intake (type and amount of foods), food preferences, feeding behaviour and growth and self-reported maternal feeding practices and parenting practices and efficacy. Covariates will include sociodemographics, infant feeding mode and temperament, maternal weight status and weight concern and child care exposure.

**Discussion:**

Despite the strong rationale to focus on parents' early feeding practices as a key determinant of child food preferences, intake and self-regulatory capacity, prospective longitudinal and intervention studies are rare. This trial will be amongst to provide Level II evidence regarding the impact of an intervention (commencing prior to age 12 months) on children's eating patterns and behaviours.

**Trial Registration:**

ACTRN12608000056392

## Background

Around two-thirds of Australians are overweight or obese and, in 2008, the total cost of obesity (excluding overweight) in Australia was $ 8.3 billion [1]. Primary prevention of childhood overweight is a high priority given 20-25% of Australian 2-8 year olds are already overweight [2,3] and at substantially increased risk of becoming overweight adults, with attendant increased risk of morbidity and mortality [4,5]. There is also correlational evidence linking parenting style and early feeding practices to child weight status [6] but prospective longitudinal or intervention studies are rare.

### Why target early feeding practices?

Parents, particularly mothers, are the 'gate keepers' of children's eating environments [7]. Parent early feeding practices (i) determine infant exposure to food (type, amount, frequency) and (ii) include responses (e.g. coercion) to infant feeding behaviour (e.g. food refusal). These feeding practices strongly influence children's eating patterns, which are firmly established by five years of age and lay the foundation of adult eating habits [8,9]. The degree of parental control over early feeding (restriction, monitoring and pressure) has been associated with child eating behaviour (preferences and intake) and weight status [6,8]. Rapid early weight gain before two years of age is associated with a 2-3 fold increase in risk of later overweight [10,11]. Most excess weight gained before puberty is gained by the age of five years (91% girls, 70% boys) [12]. Contemporary feeding practices are seen to stem from culture, tradition and family experience. They evolved in the context of relative food scarcity (less than 2-3 generations ago) and have not adapted to western environments, where excess food can pose a major health risk. Therefore, new approaches that reflect key contemporary determinants of child eating behaviour are required [7].

Given that poor eating patterns emerge early in life, early-life interventions are required. Recent US, nationally representative, cross-sectional data from the Feeding Infants and Toddlers Study (FITS) [13] (n = 3022, 4-24 months), report poor intakes of fruit and vegetables and frequent use of non-core foods [14]. Webb et al. [15] report similar dietary quality issues in 429 Australian children aged 16-24 months who were enrolled in an asthma prevention trial. Approximately half the children drank cordial daily and two-thirds consumed fried potato, confectionary and non-milk sweetened beverages at least once over the three day record period. The mean consumption of 'extras' foods (energy-dense, nutrient poor) was 157 g per day and contributed 27% of daily energy intake.

### Approaches to improving eating patterns in preschoolers: the evidence gap

A 2008 review [6] examining the role of parenting and feeding practices in child eating behaviour and weight status highlights the explosion of research interest in this area. It concluded that (i) the vast majority of evidence is cross-sectional or experimental from a 'quasi laboratory' setting (only 7 of 67 studies were longitudinal, none of which included children under 5 years); (ii) very few studies examine parent feeding practices, child eating behaviour, intake and weight as a multidirectional mediation model; (iii) only two studies (both in preschools) examined whether parent feeding practices can be modified; and (iv) most studies failed to evaluate covariates, particularly maternal weight status and family socioeconomic status [6].

Only two intervention studies have evaluated the influence of parenting and feeding practices on child eating behaviour and weight status. NEAT [16] used a quasi-experimental design to evaluate a 6-month home-visit program to enhance feeding practices in toddlers (n = 135; mean age 19 months at baseline) and found minimal effects. This study was limited by a non-randomised design, use of a convenience sample, a short time-frame and absence of direct outcome data on child weight status, food preference or intake. Further, the intervention may have been too late to change already-established eating patterns. The second was a pilot study [17] that involved a 16-week home-visit intervention for 40 Native American families (children aged 9-36 months). At the end of the program there was a reduction in weight-height z-score in active versus control groups (mean -0.27 ± 0.31; *P *= 0.06).

The NOURISH randomised controlled trial (RCT) is designed to promote feeding practices that will support healthy weight and growth. It will provide impact evaluation with respect to improving feeding practices and infant food preferences and intake up to age two years, as potentially modifiable determinants of weight status. It is intended that weight status at five years of age will be the primary outcome in longer follow up of the cohort, subject to further funding.

### Rationale for the proposed NOURISH intervention

**Figure 1:**
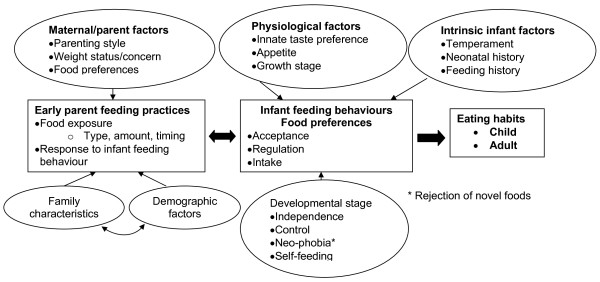
**Key factors that influence the reciprocal relationships between parent feeding practices and infant feeding behaviour**.

While parents and infants share a common genetic propensity for weight gain, the early feeding environment is critical for establishing eating habits [18,19]. Figure 1 summarises key factors that influence the reciprocal relationships between parent feeding practices and infant feeding behaviour, child food preferences and early food intake patterns. These, in turn, lay the foundation for later eating habits [7,8,18]. The NOURISH intervention reflects the key determinants of healthy eating behaviour in infants and children.

### Exposure and acceptance

Neophobia, the rejection of novel foods, is a normal adaptive response, but is readily modified by experience, particularly familiarity arising from repeated exposure [8]. Repeated (≥ 10) neutral exposures within a short time frame enhance acceptance of new foods; both healthy (eg fruit and vegetables) [8,19] and unhealthy (high fat and/or sugar, low nutrient) foods [20]. Unfortunately, the wide availability of the latter energy dense, low nutrient foods ('non-core' or 'extras') in family diets means even very young children have high levels of exposure, potentially enhancing their access to and preference for such foods [7]. There is a dearth of food intake data from Australian children under two years. Our pilot study of a random sample of 361 mothers of toddlers (aged 12-36 months) found evidence of poor dietary quality. On the day of survey 30% consumed ≥ 2 non-core foods and 39% had sweetened drinks.

### Self-regulation of intake

Self regulation of intake in response to internal hunger and satiety cues is innate in infancy, but easily overridden by social and emotional cues from adults [21]. Parental feeding practices such as explicit encouragement and praise, coercion, coaxing and the use of alternatives or rewards (food or otherwise) have been shown to be ineffective in improving food intake and variety [8,22]. Satter argues for a *'parent provide child decide' *approach where the parent is responsible for providing safe, nutritious, developmentally-appropriate food and the child decides if, and how much to eat [22]. However, data from our pilot study (see above) showed that such an approach is uncommon: 75% of mothers self-reported coaxing or coercing their child to eat more; only 56% interpreted general food refusal as satiety and 40% at least sometimes used food as a reward. More mothers were concerned about their child being underweight (22%) than overweight (9%) [23]. These data are consistent with results from FITS [24,25] and indicate a concerning prevalence of maternal anxiety about feeding, use of non-neutral approaches to food refusal, emotional use of food and failure to appropriately respond to internal hunger and satiety cues.

### Attachment and parenting skills

Attachment refers to the enduring emotional tie between an infant and their primary caregiver [26] who share repeated, characteristic interactions that shape each others' behaviour. Secure attachment develops when care is consistent, warm and sensitive [27]. Enhancing attachment is a common goal of early intervention and prevention programs to promote parenting competence and skills and child health and well being [27,28]. A meta analysis [28] of 88 interventions (n = 1503), concluded that brief behavioural interventions (with 5-16 versus more than 16 sessions), that start in mid-infancy rather than perinatally, are most effective in enhancing maternal sensitivity (appropriate and prompt emotional and verbal responses to infant signals). Sample characteristics (SES, multiple social risk factors, adolescent mother, prematurity) were not effect modifiers.

While the attachment paradigm has not been used directly in the nutrition promotion context, attachment interventions commonly use video taping of feeding sessions as an intervention strategy and/or outcome measure [28,29]. However, given that maternal sensitivity to infant cues of hunger and satiety are central to positive feeding practices, attachment provides a highly plausible and novel framework within which to develop behavioural strategies to enhance parental competence and skills in early feeding.

Parenting styles can be defined on the dimensions of behavioural control and responsiveness (warmth) and are related to parenting behaviours and feeding practices [6]. In a cross-sectional study of 4-year olds (n = 231), authoritative parenting and feeding styles (high control, high warmth) were independently associated with higher intakes of dairy foods and vegetables, whilst authoritarian styles (high control, low warmth) were associated with lower intake of vegetables [30]. A prospective study of 5-year olds (n = 872), reported that those exposed to authoritarian parenting were five times more likely to be overweight two years later than those exposed to authoritative parenting practices (after adjustment for a range of covariates, including child weight) [31]. Several authors recommend targeting parenting and feeding styles, specifically encouraging authoritative feeding, in interventions to prevent child overweight [6,30,31].

**Anticipatory guidance **is a proactive and preventive approach. It provides parents with information about behaviours they can expect and positive ways to manage these, rather than waiting until parents seek advice once problems have become established. This approach has been shown to be effective in improving family and child outcomes across a range of domains [32].

Overall, the following problems appear to be prevalent for Australian infants and toddlers: high exposure to non-nutritive, energy dense foods; maternal concern about feeding; use of non-neutral approaches to food refusal; emotional use of food; and coercive maternal feeding practices that fail to respond appropriately to infant hunger and satiety cues. These practices are linked to increased obesity risk [6] and their reduction is the focus of the NOURISH intervention modules.

### Aims and hypotheses

The NOURISH study aims to implement and undertake impact evaluation of a community-based intervention for first-time mothers of infants aged 4-7 months at enrolment that will

(i) foster healthy food preferences, dietary intakes and eating behaviours in very young children;

(ii) initiate and maintain positive maternal feeding practices in very young children; and

(iii) enhance maternal efficacy (knowledge, skills, confidence) with respect to child feeding.

A RCT will compare self-directed access to 'usual child health services' (control) with participation in a structured, comprehensive, maternal education and peer support program delivered when the infants are 4-7 months and 13-16 months of age and which will provide anticipatory guidance to improve early feeding practices (intervention). Follow up will be at two years of age. It is anticipated the intervention will result in:

H1: increased infant/child preferences for, and intake of, fruit and vegetables (frequency and variety);

H2: reduced infant/child preferences for, and intake of, non-core (low nutrient, energy dense) foods;

H3: increased frequency of maternal feeding practices that recognise and respond appropriately to infant cues of hunger and satiety and that support infant/child self-regulation of intake; and

H4: improved maternal efficacy and confidence with respect to child feeding.

## Methods

### Overall study design

NOURISH is a multi-site RCT to be conducted in Brisbane and Adelaide, Australia. A consecutive sample of first-time mothers with healthy term infants will be recruited from postnatal wards of major maternity hospitals in both cities. Assessments and intervention will commence when the infants are 4-7 months old and will be conducted at existing child health clinics. Randomisation is to be on an individual dyad basis, stratified by assessment clinic. Follow-up will be for 18 months to 22-25 months of age. The intervention comprises two consecutive modules, each with six fortnightly group sessions, followed by monthly maintenance contacts for six months. Controls will have self-directed access to services at child health clinics. These are similar in both cities and may include growth measurements, written and web-based materials, a telephone help line and, in some cases, individual appointments (limited due to staff availability).

Funding for this first phase is from the National Health and Medical Research Council (Grant No 426704). Further funding is being sought to extend follow-up to five years to assess sustainability of impact on modifiable determinants of eating behaviour and to add outcome evaluation of effect at five years of age on weight status and other nutritionally-related outcomes (e.g. oral health). Ethical approval to conduct the study has been obtained from eleven Human Research Ethics Committees across both sites (Queensland University Technology HREC 00171 Protocol 0700000752.)

### Recruitment and participants

**Figure 2:**
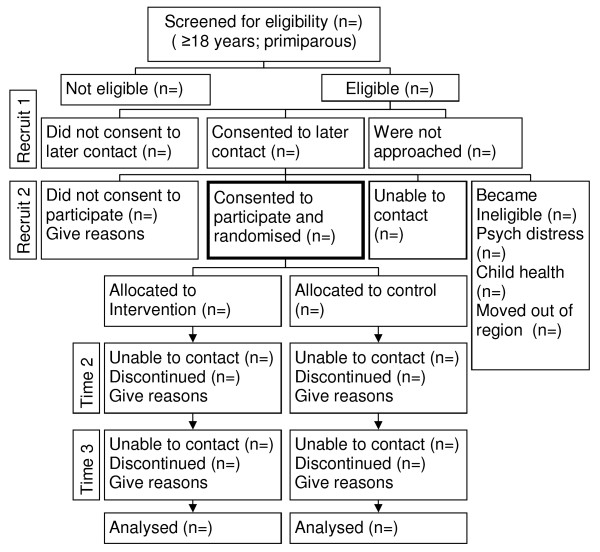
**Study design**.

Recruitment will be a 2-phase process and is summarised in Figure 2.

#### Recruit 1

The sampling frame is all first-time mothers delivering healthy term infants at three major public maternity hospitals in Brisbane (Royal Brisbane Women's Hospital, Logan Hospital and Mater Hospital) and Adelaide (Flinders Medical Centre, Lyell McEwin and Children, Youth and Women's Health Service) over a consecutive 4-month period. Eligible mothers will be approached within 72 hours of delivery for consent to be contacted again regarding full enrolment in the study when their infant is 4-7 months. Recruitment in two cities is required to provide sufficient participants within the 3-year grant time frame. Written consent and contact details will be collected, plus brief demographic data from consenters and non-consenters.

#### Recruit 2

Consenting Recruit 1 mothers will be contacted again by mail three months later and sent the information sheet, consent form, two brief questionnaires, details of possible assessment clinic sites in their area and a reply-paid envelope. All those who respond, irrespective of consent decision, will be eligible for a draw of eight baby-product vouchers. Those declining consent will be asked to complete a brief questionnaire to supplement Recruit 1 data in order to collect information to assess potential selection bias. Intending participants will be asked to complete the consent form, a questionnaire to assess continued eligibility, and a form to indicate their top three preferences for assessment venues, days and times. We will make up to three attempts to telephone those who fail to return a response within two weeks. Appointments for assessment sessions will be mailed. 'No-shows' to base-line assessment will be contacted by telephone (as above) and either rescheduled or supplementary data collected.

### Selection criteria

#### Inclusion criteria

Infants must be born healthy and at term (>35 weeks, >2500 g). Mothers must have delivered this infant as their first live infant, be at least 18 years of age, willing and able to attend sessions at designated metropolitan child health clinics, and have facility with written and spoken English.

#### Exclusion criteria

Mother-infant dyads will be excluded if the infant has any diagnosed congenital abnormality or chronic condition likely to influence normal development (including feeding behaviour) or the mother has a documented history of domestic violence or intravenous substance abuse or self-reports eating, psychiatric disorders or mental health problems.

These criteria will be assessed at Recruit 1. At Recruit 2, the eligibility criteria of 'healthy baby' will be reviewed by asking mothers to check a list of specified conditions. In addition, at Recruit 2 the Kessler Psychological Distress Scale (K10) [33], a 10-item self assessment widely used in mental health surveys in Australia to screen for psychiatric morbidity, will be administered. Mothers in the clinical range will be deemed ineligible and referred to their general practitioner.

The exclusion criteria of psychiatric morbidity, domestic violence and substance abuse will identify 'at risk' mothers for whom a generic group program focussed on feeding is not likely to be appropriate and for whom exclusion avoids respondent overload. Enrolment at 4-7 months is supported by our pilot study data and will facilitate maternal engagement based on some early feeding experience, but before feeding dynamics are entrenched. First-time mothers will be selected to minimize difficulties in regulating exposure and implementing the intervention in conjunction with feeding older siblings.

### Allocation

On receipt of signed consent and completion of baseline assessment, participants will be randomly allocated to intervention or control according to a permuted-blocks randomisation schedule generated by the Institute's Research Methods Group, which includes this study's statistician, all of whom will otherwise not be involved in data collection or intervention delivery. Block sizes of four within strata defined by location of assessment clinic will be generated. Participants will be notified by mail of allocation and appropriate program schedules.

### Intervention

#### Process

Fortnightly group sessions (n = 10-15 mothers/primary carers per group), will be delivered at existing child health clinics and co-led by a dietitian and psychologist with paediatric experience. Delivery through existing child health infrastructure will enhance cost effectiveness and relevance, build staff capacity, facilitate dissemination and translation of findings into practice and provide participants with convenient access in their local area. Onsite child care will be provided. Strategies to maintain intervention fidelity will include use of a standardised facilitator manual including protocols, procedures, activities and materials for each group session and standardised participant materials. Facilitators will meet regularly by teleconference with the study coordinators for session planning, review and supervision.

#### Content

The emphasis for parents will be on healthy eating, feeding relationships and healthy growth, rather than obesity prevention. The content will be consistent with recommendations made by Birch [8,18] and Satter [19,34] and informed by our pilot work. Modules are timed to provide anticipatory guidance and start when infants are aged 4-7 and 13-16 months. Module 1 will focus on establishing solid feeding including variety and texture, neutral repeated exposure to healthy foods, neutral limited exposure to non-core foods and realistic expectations of the growth and nutritional requirements of healthy infants. Module 2 will promote development of a positive feeding environment and managing toddler eating behaviour in the context of increasing autonomy and transition to eating with the family and in wider social settings. It encourages a structured food choice and eating pattern, positive role modelling and avoidance of coercion, use of rewards and emotional feeding.

Both modules promote authoritative parenting practices and feeding styles [30,31] (high control and warmth); maternal recognition of and trust in child cues of hunger and satiety; and consistent, responsive use of developmentally-appropriate structure and limits. Group sessions are interactive and include a range of strategies consistent with a cognitive behavioural approach to enhance maternal self-efficacy and to build supportive environments (e.g. information pack for family members and other carers, including child care providers). All intervention participants receive a workbook to ensure optimal intervention dose, monitor strategies attempted at home, and to encourage retention. In addition, those unable to continue with sessions receive an early feeding text by Satter [22] designed for parents. Fridge magnets with the key message from each module will be provided. Mothers participating in the second intervention module will be offered onsite child care provided by adjunct care providers.

Physical activity will not be targeted in the intervention package as at the time of planning the National Physical Activity Guideline [35] did not go below five years of age. Moreover, there is no evidence that physical activity would influence food preferences or maternal response to hunger or satiety cues and there are no validated tools to measure activity outcomes in this age group. However, avoidance of television watching whilst feeding will be discouraged. Innate variability in activity should be controlled for by randomisation.

### Measurements and procedures

#### Outcome measurements

Outcome variables are described in detail in Table 1 and along with covariates are to be measured by mother-completed questionnaires at baseline (Time 1, age 4-7 months) and six months after the completion of each 12-week intervention module at age 13-16 months (Time 2) and 22-25 months (Time 3). Questionnaires are to be completed at home and brought to assessment clinics where maternal and infant weight and height/length will be measured. Child intake will be measured at Times 2 and 3 using a telephone 24-hour recall conducted by a dietitian and a 2-day (one week and one weekend day) food record completed by the mother. Randomisation should optimise chances of no group difference in Time 1 infant intake which, along with maternal intake, will not be collected due to the resource and participant burden implications. Assessors will be trained and will not be involved in intervention delivery. Where a number of assessment tools are not suitable across all age groups, we have selected for age appropriateness within constructs, rather than consistency across time points.

**Table 1:** Outcome measures for the study

**Participant**	**Measure - assessment sessions at child health clinics**	**T1**	**T2**	**T3**
**Infant/Child**				

Food intake records H1	3 non-consecutive days (including weekend day), using 2 × 24 h food records + telephone 24 h-recall. Standard protocol (including estimation of breast milk intake and standardised visual aids for serve size estimation) will match FITS [48] and other [49] studies; well accepted in pilot study.		✓	✓

Food preference H2	The Wardle tool [37] adapted to Australian target foods. Mothers rate on 5-pt scale from 'likes a lot' - 'dislikes a lot' with option for 'hasn't tried it'	✓	✓	✓

Feeding behaviour H3	Children's Eating Behaviour Questionnaire [50]. Validated 35-item parent report of satiety responsiveness, fussiness, food responsiveness, enjoyment, emotional over/under eating.		✓	✓

Weight & growth	Recumbent length and weight. Weight, length and weight-for-length z-scores calculated using CDC EpiInfo (version 3.3.2).	✓	✓	✓

**Maternal**				

Feeding style and practices H3	The Infant Feeding Questionnaire [36]. 20-item - under/over-eating, hunger, infant cues, scheduling, use of food to calm.	✓	✓	
	Child Feeding Questionnaire [43] 28-item - 2-11 yrs. - feeding attitudes, practices, perceptions/concerns regarding weight.			✓

Parenting skills H4	Four brief scales from LSAC measuring warmth, irritability, consistency and overprotection (24 items)[41]	✓	✓	✓

BMI	Height and weight using standard procedures	✓	✓	✓

**T1 **= baseline, pre-allocation, infants 4-7 mths; **T2 **= mid study- 9 mths, infants aged 13-16 mths; **T3 **= final -18 mths, infants aged 22-29 mths. LSAC = Longitudinal Study of Australian Children

#### Covariates

A comprehensive range of sociodemographic, maternal and infant covariates will be collected. At the first contact (ie at birth) data collected on the larger eligible sample will include maternal age, education, ethnicity, marital status, household composition, self-reported pre-pregnancy weight status, perceived level of support with parenting, lifestyle (smoking and alcohol intake) and health problems (diabetes, preeclampsia) during pregnancy, birth weight and breast feeding intentions. Baseline assessments (Time 1) of those consenting to full enrolment will include maternal mental health, current breast/bottle/solids feeding (also collected from non-consenters), family income, parental employment, child care use, child health issues, maternal diet, activity, smoking and alcohol intake. Data to be collected at subsequent assessments are maternal lifestyle behaviours (e.g. activity and fruit and vegetable intake) and any demographic data that are likely to change (including marital status and birth of subsequent children).

Maternal covariates will be assessed at Times 1 and 3. These will include maternal body mass index and baseline infant feeding practices, attitudes and beliefs assessed using the 20-item Infant Feeding Questionnaire [36]. Maternal food preferences influence foods made available to the child and hence child food preferences, and will be described using the Wardle tool [37] (Table 1) with an additional option 'like but don't usually eat' (based on our pilot study feedback that this addition was warranted). Maternal concerns regarding their own weight and eating-related issues influence child feeding practices [38]. Maternal restrained eating will be determined using the Restraint Scale, a validated, widely used 21-item scale [39]. The 5-item Weight Concern Scale [40] will assess maternal perceptions regarding their own weight gain, body weight and shape.

Child covariates assessed will include detailed data on early infant feeding (breast, bottle, type of formula, exclusive breast feeding, use of other fluids, age of introduction of solids), early growth rate from birth to baseline, temperament and child care experience.

To enable comparisons with normative Australian data, NOURISH will use demographic, parenting, child temperament and child care measures that were developed and validated for the nationally-representative Longitudinal Study of Australian Children (LSAC) [41].

### Process evaluation

Process evaluation will include facilitator self-ratings of quality of group facilitation, content fidelity and group processes for every session. One session for each group will be rated for quality and fidelity by an independent experienced observer using a standardised process [42]. Participant satisfaction will be assessed by questionnaire at completion of each module and detailed attendance records will be kept to quantify 'dose' of intervention received.

### Sample size

Based on the number of eligible births in the target hospitals, a four month recruitment period and an overall baseline participation rate of 42% (60% Recruit 1, 70% Recruit 2) we aim to recruit 820 participants. Assuming a 65% completion rate, approximately 265 per intervention arm will available for the final analyses. There are no data published on the likely intra-cluster correlation coefficients (ICC) for the outcomes of our study, but we anticipate that they will be moderate given the (likely) greater demographic and socio-economic similarity within parents/carers attending the same clinic compared to those attending different clinics, and that the outcomes we are considering are associated with these characteristics [43]. A recent New Zealand study [43] reported a median ICC of 0.09 for nutrient outcomes obtained from 24-hour recall data in a cluster sample aged 1-14 years. In the absence of more direct data, we have assumed conservatively an ICC of 0.10 and, for an average cluster size of 20, we anticipate a design effect of the order of 2.9. Hence our sample size of 265 per group is effectively a sample size of 92 per group. For this, we shall be able to detect, with 80% power and type I error of 5% (two-tailed), meaningful clinical differences in prevalence of outcomes (indicator behaviours for positive feeding practices) as noted in Table 2.

**Table 2:** Minimum meaningful differences between control and intervention groups, and those detectable with 80% power, 5% significance (two-tailed) for endpoint sample size of 265 per group, assuming a design effect of 2.9.

**Intake**	**Prevalence #**	**Detectable**	**Behaviour**	**Prevalence #**	**Detectable**
Fruit^a^	82 vs ≥ 95%^(iii)^	82 vs ≥ 95%	Offer new food >10 times^b^	28 vs ≥ 75%^(iii)^	28 vs ≥ 48%

Vegetable^a^	67 vs ≥ 95%^(iii)^	67 vs ≥ 84%	Refuses food- assume not hungry, take food away often/very often/always^b^	56 vs ≥ 84%^(i)^	56 vs ≥ 75%

Salty snacks^a^	27 vs ≤ 17%^(ii)^	27 vs ≤ 11%	Refuses food-offers no replacement food often/very often/always^b^	29 vs ≥ 44%^(i)^	29 vs ≥ 49%

Sweet beverages^a^	44 vs ≤ 28%^(ii)^	44 vs ≤ 25%	Use food as reward 'hardly ever'^b^	55 vs ≥ 83%^(i)^	55 vs ≥ 74%

Fried potato^b^	17 vs ≤ 8%^(i)^	17 vs ≤ 5%	Insist child eat 'hardly ever'^b^	46 vs ≥ 69%^(i)^	46 vs ≥ 66%

# Prevalence = proportion of children consuming food on day of record - Anticipated and *a priori *defined meaningful differences in control versus active groups; Control prevalences are based on descriptive cross-sectional data from (a) FITS[14] or (b) our pilot study for infants 19-24 or 12-36 months respectively. Criteria to estimate expected differences (in direction of desirable intake/behaviour) were (i) relative increase/decrease of 50% (ii) equivalent to intake at 9-12 months or (iii) increase/decrease to optimal.

Our definitions of meaningful differences are based on detecting a difference between the control and intervention groups at study end (age 22-25 months) in prevalence of intake of key foods indicative of dietary quality and of key parent practices that support self-regulation. We are also assuming that randomisation will be successful at baseline and that attrition is random across the groups. These assumptions will be quantified at the point of analysis. As our analytical approach will consider all available data, these sample size calculations based only on end-point data will tend to under-estimate our power, all other assumptions holding. We shall have sufficient power for our primary outcomes with the exception of specific intake of fried potato, salty snacks, and sweet beverages, where power will be lower, but still moderate.

### Data analyses

Due to expected changes in feeding behaviour and intake over 18 months, group but not time effects will be examined at each time point, with the exception of growth. Primary analysis will be according to intention-to-treat principles. A generalised estimating equations analytical approach will be used to account for the clustering within assessment clinics, as well as to permit data to be included for those not completing all assessments (thus optimizing power). Success of randomisation will be considered based on a comparison of the two groups across a range of centre, child, and carer characteristics, against *a priori *defined meaningful differences. Any noted imbalances at baseline will be accounted for in multivariable logistic regression modelling, adjusting for their potential confounding effect on the impact of intervention on each outcome.

## Discussion

Given that only 30% of eligible mothers are expected to complete the trial, there is potential for selection bias, and threats to generalisability. It is important to note that the evidence that currently informs early feeding advice is cross-sectional, observational or quasi-experimental with inherent selection bias. Thus, despite potential selection and retention bias, this study will represent a major advance in understanding the feasibility and impact of a structured, comprehensive feeding practices intervention with first-time mothers. Once we demonstrate efficacy, then further research will be required to determine effective strategies to access and engage hard-to-reach groups. The study will demonstrate intermediate behavioural outcomes and justify extending follow up to directly evaluate obesity risk outcomes. The recruitment strategy is designed to provide a comprehensive, representative sampling framework and reduce the selection bias inherent in a volunteer sample. The target public hospitals cover 70% of Brisbane metropolitan births and 50% of births in South Australia and should provide a broad demographic profile. An important strength of the study will be our capacity to quantify bias by characterising the study sample in comparison with the broader source population, based on the detailed non-participant response data at Recruit 1 and 2, including reasons for non-consent.

The NOURISH trial addresses a major public health problem and is consistent with current government and community foci on early childhood as the foundation for life-long health and well being [44]. Existing trials evaluating both prevention and treatment of obesity in young children have demonstrated limited outcomes, at least in part due to design and methodological issues [45-47] Given that very few intervention studies include children under two years of age, it may also be that interventions have started too late, after feeding and eating patterns have been established and are more difficult to modify. Additional plausible rationales for very early interventions are that there is evidence of poor dietary quality even in very young children [14], rapid weight gain before two years is a risk factor for later overweight [10] and parents may be more amenable to advice and behaviour change that targets their new and, perhaps particularly their first, baby. Despite the strong rationale for early intervention, quality evidence to guide strategies to improve eating patterns, prevent overweight or promote healthy weight in very young children is extremely limited.

The NOURISH trial will be amongst the first to provide Level II evidence of the impact of a comprehensive, structured intervention to promote positive parent feeding practices on very early child food intake and preferences. It also has the potential to provide detailed descriptive prospective data to extend our understanding of the complex reciprocal and synergistic relationships between parenting and feeding practices and child feeding behaviour and weight status and the modifying effects of socio-demographic, infant and maternal covariates.

## Competing interests

The authors declare that they have no competing interests.

## Authors' contributions

LD took the leading role in designing the study, writing the grant that was subsequently funded by the National Health and Research Council and modifying the grant for publication. AM, DB and JN contributed to the study design and grant preparation. AF, GD and GC provided expert input and support for preparation of the grant proposal with particular emphasis on clinical issues, selection criteria, recruitment strategies and ethical issues. All authors read and approved the final manuscript.

## Pre-publication history

The pre-publication history for this paper can be accessed here:


